# A triad of NRP2, DLX and p53 proteins in lung cancer metastasis

**DOI:** 10.18632/oncotarget.22097

**Published:** 2017-10-26

**Authors:** Harry A. Drabkin, Julia Starkova, Robert M. Gemmill

**Affiliations:** Harry A. Drabkin: The Hollings Cancer Center and Medical University of South Carolina and Ralph H. Johnson VA Medical Center, Charleston, SC, USA

**Keywords:** neuropilin, receptor tyrosine kinases, TGF beta, epithelial-mesenchymal transition, semaphorin 3

Efforts to identify Receptor Tyrosine Kinases that drive tumor growth have not extended to co-receptors. Neuropilin co-receptors (NRP1/2) have critical roles during development and altered expression is frequent in cancer [1]. Two recent papers from the Drabkin / Gemmill and Xiao laboratories demonstrate that NRP2, the high-affinity receptor for SEMA3F, plays an essential role in lung cancer invasion, metastasis and acquired drug resistance [2, 3]. These and other studies indicate that NRP2 is a key component in tumor progression pathways involving TGFß, p53 and DLX2, suggesting it may be an important therapeutic target.

As primary receptors, NRP1 and NRP2 were shown to bind secreted class 3 semaphorins (Sema3s), which have a repulsive role in axonal guidance and suppress signaling via AKT, STAT and MAPK pathways. In contrast, both NRPs enhance phosphorylation and downstream signaling when interacting with VEGF and other heparin-binding growth factors.

The Drabkin / Gemmill team discovered that a previously uninvestigated isoform, NRP2b, bearing little similarity to the prototype receptor NRP2a in its cytoplasmic domain, was required for pro-tumorigenic activities of TGFß in lung cancer cells [3]. Their previous studies had shown that total NRP2 was upregulated by TGFß, while NRP1 levels were unchanged or downregulated [4]. Subsequent work demonstrated that NRP2b was preferentially upregulated by TGFß and its expression enhanced migration, invasion, metastasis, tumorsphere formation and acquired EGFR inhibitor resistance associated with EMT features [3]. In contrast, NRP2a expression had reduced or inhibitory tumorigenic effects compared to NRP2b. Although previous studies found that NRP1 functioned as a co-receptor for TGFß with effects on SMAD activation [5], NRP2 affected non-canonical signaling in lung cancer cells [4]. NRP2b was found to specifically enhance phospho-AKT levels by hepatocyte growth factor, which was upregulated in TGFß-treated cells [3]. Also, unlike NRP2a, NRP2b failed to interact with GIPC1, a PDZ-domain scaffold protein that regulates receptor endocytosis and trafficking. Thus, differential receptor trafficking may underlie signaling differences between isoforms.

Work from the Xiao laboratory found that NRP2 upregulation was linked to p53 hot spot mutations, which have been associated with increased migration and metastasis [2]. *In vitro*, p53 mutant lung cancer cells underwent an epithelial-mesenchymal transition (EMT) with increased migration, invasion and downregulation of the homeodomain gene, DLX2. Of note, DLX2 knockdown induced EMT and NRP2 levels, whereas forced DLX2 expression caused cells to grow in a clustered epithelial appearance. Furthermore, knockdown of NRP2 led to reduced expression of mesenchymal markers, reduced cell migration and impaired lung colonization by p53 mutant cells. As was previously shown in neurons, DLX2 overexpression in p53 mutant lung cancer cells downregulated NRP2 levels.

DLX proteins have previously been linked to TGFß and p53 pathways. In early tumor development, TGFß acts as a suppressor, whereas at later stages it enhances progression through its effects on invasion, metastasis and tumor stem cells. Enhanced growth factor signaling with DLX1/2 upregulation is one mechanism of overcoming TGFß-mediated growth suppression. In leukemia, Starkova reported that DLX1 and 2 were upregulated by FLT3 activation, which in turn inhibited canonical TGFß responses [6]. The mechanism involved ERK / JNK activation and upregulated DLX expression, which reduced nuclear phospho-SMAD2. As anticipated, FLT3 inhibition sensitized cells to TGFß-mediated growth suppression, suggesting that midostaurin (PKC412) and similar agents used in the clinic may exert part of their anti-leukemic activity by this mechanism.

In murine NMuMG breast cells, TGFß was shown to upregulate Dlx2, which protected them from TGFß-induced growth inhibition and apoptosis [7]. This involved upregulation of the EGFR ligand, betacellulin (BTC), with increased downstream signaling. Of relevance to lung cancer, increased BTC expression was identified in damaged bronchial epithelia and lung cancer cell lines were shown to respond to BTC by increased CXCL8 (IL-8) production. Furthermore, during EMT, BTC levels were increased by ZEB1-mediated repression of miR-200, resulting in an EGFR autocrine loop.

In addition to suppression by mutant p53, DLX2 was identified in a screen for novel suppressors of senescence. Confirmatory studies demonstrated that DLX2 prolonged lifespan and reduced senescence-associated ß-galactosidase. Moreover, activated p53 was downregulated and traced serially to hierarchical effects of ATM / ATR proteins, other members of the PI3 kinase-related kinase family, DNA-PK, mTOR and SMG1, and their upstream regulator, the Triple T Complex, which recruits HSP70 / 90 chaperones for folding [8]. Of note, in a large series of breast cancer patients, DLX2 overexpression was mutually exclusive with p53 mutations, supporting their role in a common pathway.

In summary, NRP2 is a common upregulated target of TGFß-mediated EMT and p53 mutations, which result in enhanced migration, invasion, metastasis and drug resistance. Whether mutant p53 preferentially upregulates NRP2b is an important unanswered question. Interestingly, while TGFß upregulates NRP2, it suppresses its repulsive ligand, SEMA3F. Similarly, since SEMA3F is upregulated by wild type p53 while NRP2 is suppressed via DLX2, p53 mutations result in the same inverse ligand / receptor responses, ostensibly to facilitate migration. DLX proteins are involved in these responses and DLX2 overexpression provides an alternative to p53 mutation. Thus, further study of these interacting pathways is likely to be rewarding.
